# Ultrasound-Guided Transversalis Fascia Plane Block Versus Transversus Abdominis Plane Block for Postoperative Analgesia After Repeat Cesarean Delivery: A Prospective Observational Study

**DOI:** 10.3390/healthcare14172682

**Published:** 2026-08-24

**Authors:** Taha Emre Ötügen, Fethi Akyol, Talha Karataş, Gamze Ataş, Hakan Gökalp Taş, Ufuk Kuyrukluyıldız

**Affiliations:** 1 Department of Anesthesiology and Reanimation, Mengucek Gazi Training and Research Hospital, 24100 Erzincan, Turkey; tahaemreotugen@gmail.com (T.E.Ö.); dr.gamzeatas@gmail.com (G.A.); 2 Department of Anesthesiology and Reanimation, Faculty of Medicine, Erzincan Binali Yildirim University, 24100 Erzincan, Turkey; fethi24@windowslive.com (F.A.); drufuk2001@gmail.com (U.K.); 3 Department of Anesthesiology and Reanimation, Kovancilar State Hospital, 23850 Elazığ, Turkey; talha1905-74@hotmail.com

**Keywords:** cesarean section, transversus abdominis plane block, transversalis fascia plane block, postoperative pain, regional anesthesia, ultrasound-guided block

## Abstract

Background: Effective postoperative pain control after cesarean delivery is essential for facilitating early mobilization and reducing analgesic requirements. Ultrasound-guided fascial plane blocks, such as the transversus abdominis plane (TAP) and transversalis fascia plane (TFP) blocks, are increasingly used as components of multimodal analgesia; however, direct comparative data between these techniques remain limited. Methods: In this prospective observational study, 90 patients undergoing repeat cesarean deliveries under spinal anesthesia were administered either a bilateral ultrasound-guided transversus abdominis plane (TAP) block or a transversus fascia plane (TFP) block in accordance with standard clinical practice. The intensity of postoperative pain, assessed both at rest and during movement, was determined using VAS scores at 3, 6, 12, and 24 h after surgery. The time until first ambulation and additional postoperative analgesic consumption beyond the standardized baseline analgesic regimen over the first 24 postoperative hours were also evaluated. Results: The patients who were administered a TFP block had diminished postoperative pain levels, reduced additional postoperative analgesic requirements, and expedited mobilization relative to those administered a TAP block. The TFP group had considerably lower movement-related pain levels within the first 24 h following surgery. Opioid-related adverse effects were comparable between groups. Conclusions: After a repeat cesarean delivery, applying a TFP block was linked to better postoperative analgesia and fewer analgesic requirements than applying a TAP block. These findings suggest that TFP blocks may represent an effective component of multimodal analgesia in obstetric anesthesia.

## 1. Introduction

Cesarean delivery is among the most frequently performed surgical procedures worldwide. Postoperative pain following cesarean section may adversely affect maternal recovery by delaying early mobilization, interfering with breastfeeding, and impairing mother–infant bonding [1,2,3,4]. Moreover, inadequate pain control has been associated with delayed functional recovery, increased opioid requirements, and a higher incidence of opioid-related adverse effects. Therefore, effective multimodal analgesia remains a cornerstone of Enhanced Recovery After Surgery (ERAS) pathways for cesarean delivery [5,6].

Ultrasound-guided fascial plane blocks are increasingly being incorporated into multimodal analgesic strategies after cesarean delivery [5,7,8]. One of the most popular methods for postoperative analgesia after a Pfannenstiel incision is the transversus abdominis plane (TAP) block [7,9]. Accumulating evidence suggests that a TAP block can improve postoperative analgesia and decrease opioid requirements after cesarean delivery [9,10]. However, the location of injection and the local anesthetic’s distribution within the fascial plane may affect the analgesic’s effectiveness [11,12].

A new alternative truncal block that targets the lower thoracic–upper lumbar nerve branches and the thoracolumbar fascial plane is the transversalis fascia plane (TFP) block [13,14]. A TFP block may offer wider coverage of the T12–L1 dermatomes implicated in Pfannenstiel incision analgesia and a more proximal distribution of local anesthetic than a TAP block [11,12,14]. Preliminary studies have suggested that TFP block may improve postoperative analgesia after cesarean delivery. The first clinical experience reported effective and prolonged postoperative analgesia following bilateral TFP block, supporting the feasibility of this technique in cesarean delivery. Subsequently, Rahimzadeh et al. compared TFPB with TAPB in women undergoing elective cesarean delivery and found comparable postoperative pain scores, analgesic consumption, and time to first analgesic request between the two techniques. More recently, Baghirzada et al. demonstrated that TFPB reduced postoperative opioid consumption and pain scores compared with placebo in women receiving intrathecal morphine. However, direct comparative evidence between TFPB and TAPB remains limited, and data specifically evaluating women undergoing repeat cesarean delivery are lacking. Therefore, further comparative studies are warranted to clarify the relative effectiveness of these two fascial plane blocks in this patient population [13,15].

There is also a dearth of direct comparative data regarding TAP and TFP blocks in patients undergoing repeat cesarean deliveries, especially with respect to postoperative pain trajectory, mobilization outcomes, and analgesic consumption [14,15]. Additionally, relative to women undergoing their first cesarean procedure, those undergoing repeat cesarean deliveries constitute a unique clinical population with potentially different postoperative pain characteristics and analgesic requirements [3,4].

The purpose of this study was to compare the mobilization results, analgesic usage, and postoperative pain levels between TFP and TAP blocks in patients undergoing repeat cesarean deliveries. We hypothesized that TFP blocks would provide superior postoperative analgesia and earlier mobilization relative to TAP blocks after a repeat cesarean delivery.

## 2. Materials and Methods

### 2.1. Study Design and Setting

Between 1 November 2024 and 1 April 2025, Erzincan Binali Yıldırım University’s Mengucek Gazi Training and Research Hospital hosted this prospective, single-center observational study. Approval from the local ethics committee was secured prior to the commencement of the study. This prospective observational study was approved by the institutional ethics committee before patient enrollment but was not prospectively registered in a public clinical trial registry. Patients undergoing elective repeat cesarean deliveries while under spinal anesthesia were included. Rather than being assigned at random, the type of postoperative fascial plane block was chosen based on anesthesiologist preference and standard clinical practices. This manuscript was prepared in accordance with the STROBE reporting guidelines for observational studies. Group TFPB comprised patients who were administered a bilateral ultrasound-guided transversalis fascia plane block, while Group TAPB comprised patients who were administered a bilateral ultrasound-guided transversus abdominis plane block. The anesthesiologist performing the block was excluded from postoperative data collection, and outcome assessments were conducted by independent anesthesiologists. All postoperative outcomes were assessed during the first 24 postoperative hours.

### 2.2. Participants

Women aged 20–45 with an ASA physical status of I or II who underwent elective repeat cesarean delivery under spinal anesthesia through a Pfannenstiel incision were considered eligible for inclusion.

The cesarean delivery number (i.e., whether the current operation was the patient’s second, third, or subsequent cesarean delivery) was recorded as an additional baseline characteristic because it may influence postoperative pain and surgical complexity.

An ASA physical status ≥III, primary cesarean delivery, general anesthesia, severe obesity (BMI ≥ 40 kg/m^2^), bleeding diathesis, preterm or post-term pregnancy, history of local anesthetic toxicity, uncontrolled gestational hypertension or gestational diabetes mellitus, refusal to participate, and inadequate or unsuccessful spinal anesthesia necessitating additional intraoperative analgesia were among the exclusion criteria.

### 2.3. Variables and Outcomes

The type of postoperative fascial plane block employed was the main exposure variable. The patients were allocated to two groups—a transversus abdominis plane block (TAPB) group and a transversalis fascia plane block (TFPB) group—according to the type of bilateral ultrasound-guided block they were administered.

Patients were prospectively enrolled as they became eligible, and block selection was determined solely by the routine clinical preference of the attending anesthesiologist, without random allocation. Enrollment continued until the prespecified target of 45 patients had been reached in each block group, at which point recruitment was terminated. Thus, the equal group sizes resulted from the predefined enrollment target and stopping rule rather than from randomization, matching, or post-enrollment balancing.

The primary outcome of this study was the postoperative pain trajectory during the first 24 h after surgery, assessed using repeated Visual Analog Scale (VAS) measurements at rest and during movement at 3, 6, 12, and 24 h.

Secondary outcomes included time to first mobilization; additional postoperative paracetamol, diclofenac sodium, and tramadol consumption beyond the standardized baseline analgesic regimen during the first 24 postoperative hours; opioid-related adverse effects, including nausea, vomiting, dizziness, and pruritus; perioperative blood loss; and perioperative hemoglobin changes.

### 2.4. Anesthetic Procedure

No sedative or anxiolytic premedication was administered before the procedure. Upon arrival in the operating room, routine anesthetic monitoring consisting of electrocardiography, noninvasive blood pressure measurement, and pulse oximetry was initiated for all participants. Hyperbaric bupivacaine was used to induce spinal anesthesia at the L3–L4 or L4–L5 interspace. No intrathecal opioids (morphine or fentanyl) were administered. Spinal anesthesia was performed using hyperbaric bupivacaine alone. All patients received 12 mg of 0.5% hyperbaric bupivacaine (Marcaine^®^ Spinal Heavy; AstraZeneca, Istanbul, Turkey) for spinal anesthesia. A bilateral sensory block at the T4 dermatome level and a Bromage motor block score of at least three prior to surgical incision were indicators of adequate surgical anesthesia.

All cesarean deliveries were performed through a Pfannenstiel incision. Before the patients were transferred to the postoperative care unit, bilateral fascial plane blocks were performed under ultrasound guidance.

### 2.5. TAP Block Technique

For the TAP block, a high-frequency linear ultrasound probe connected to a MyLab™ Six ultrasound system (Esaote S.p.A., Genoa, Italy) was positioned along the midaxillary line between the iliac crest and the costal margin. After visualization of the external oblique, internal oblique, and transversus abdominis muscle layers, the needle was advanced using an in-plane approach, and 20 mL of 0.25% bupivacaine (prepared from Buvicaine^®^ 0.5%; Polifarma, Tekirdağ, Turkey) was administered on each side into the interfascial plane separating the internal oblique and transversus abdominis muscles.

### 2.6. TFP Block Technique

For the TFP block, a high-frequency linear ultrasound probe was initially placed between the iliac crest and the costal margin and then advanced posteriorly to identify the transversalis fascia adjacent to the posterior aspect of the transversus abdominis muscle. After needle advancement under in-plane ultrasound guidance, 20 mL of 0.25% bupivacaine was administered on each side into the plane between the transversus abdominis muscle and the transversalis fascia.

Both TFPB and TAPB procedures were performed by one of several consultant anesthesiologists, each with more than five years of experience in ultrasound-guided regional anesthesia. All operators followed the same standardized institutional technique for each block.

### 2.7. Postoperative Analgesic Protocol

The first postoperative analgesic dose was administered after the patient had been transferred to her ward bed. All patients received a standardized baseline regimen of intravenous paracetamol 1 g twice during the first 24 postoperative hours, corresponding to a total scheduled dose of 2 g. According to pain intensity, additional intravenous paracetamol (1 g per dose) was administered up to a maximum total dose of 4 g within the first 24 postoperative hours. When additional non-opioid analgesia was required, intramuscular diclofenac sodium (75 mg per dose) was administered up to a maximum total dose of 150 mg within 24 h. If the VAS score remained ≥4 despite additional non-opioid analgesia, intravenous tramadol (50 mg per dose) was administered as rescue analgesia, up to a maximum of two doses during the first 24 postoperative hours. Only analgesics administered in addition to the standardized 2 g paracetamol regimen were included in the analgesic consumption analyses.

The standardized baseline paracetamol regimen was identical for both study groups. Therefore, only additional analgesics administered beyond this scheduled regimen were analyzed to compare postoperative analgesic requirements between groups.

### 2.8. Data Collection

During the first 24 h following surgery, data on demographics, perioperative features, postoperative VAS pain scores, analgesic use, mobilization time, opioid-related side effects, perioperative bleeding volume, and perioperative hemoglobin variations were recorded.

### 2.9. Bias Reduction

A number of steps were taken to enhance internal validity and reduce potential sources of bias. Only elective repeat cesarean births carried out under spinal anesthesia via Pfannenstiel incision were included in order to minimize procedural and anesthetic variability.

Anesthesiologists with more than five years of experience performing ultrasound-guided peripheral nerve blocks carried out all fascial plane blocks. Independent anesthesiologists performed postoperative evaluations; the anesthesiologist who performed the block was not involved. Throughout the trial period, all patients underwent standardized analgesic procedures and postoperative pain assessments.

### 2.10. Sample Size Calculation

To establish the minimum number of participants needed to adequately power the study, an a priori analysis was carried out using G*Power version 3.1.9.7. As repeated postoperative VAS measurements evaluated via two-way mixed ANOVA were used, 78 patients were needed to detect a medium effect size (Cohen’s f = 0.25) with an α value of 0.05, a power of 0.80, and an assumed correlation coefficient of 0.5 between repeated observations. A medium effect size (Cohen’s f = 0.25) was selected because no prior studies comparing repeated postoperative VAS trajectories between TFP and TAP blocks after repeat cesarean delivery were available to provide a reliable estimate for the effect size. Therefore, a conventional medium effect size, as conventionally recommended for repeated-measures ANOVA in G*Power, was adopted. Anticipating potential losses during follow-up, we set the final sample size to 95 participants.

### 2.11. Quantitative Variables

Postoperative VAS scores were analyzed as continuous repeated-measures outcomes obtained from serial postoperative measurements at 3, 6, 12, and 24 h. Continuous variables were summarized according to their distributional characteristics. Normally distributed data are presented as means ± standard deviations, whereas non-normally distributed variables are expressed as medians and ranges (minimum–maximum). All continuous variables were retained in their original form without categorization during the analyses.

### 2.12. Statistical Analysis

All statistical analyses were conducted using IBM SPSS Statistics for Windows (version 25.0; IBM Corp., Armonk, NY, USA). The distributional characteristics of continuous variables were evaluated with the Shapiro–Wilk test, while equality of variances was verified using Levene’s test prior to the application of parametric procedures. Categorical data are expressed as counts and percentages, whereas continuous variables are summarized as means ± standard deviations or medians (interquartile ranges), depending on the underlying distribution.

Between-group comparisons of continuous variables were conducted using either Student’s *t*-test or the Mann–Whitney U test, depending on the data distribution. Associations involving categorical variables were examined using Pearson’s chi-square test or Fisher’s exact test, as appropriate.

Postoperative VAS trajectories were primarily analyzed using mixed-design repeated-measures ANOVA to evaluate the effects of group, time, and the group × time interaction. Although unadjusted Student’s *t*-tests had initially been used for descriptive between-group comparisons at individual postoperative time points, these time-specific inferential comparisons were not part of the primary longitudinal analysis. To avoid multiplicity and ensure consistency with the prespecified longitudinal analysis, individual time-point p values are not reported. Effect sizes for repeated-measures analyses were reported using partial eta squared (η^2^p). Mean differences with 95% confidence intervals were calculated for major postoperative outcomes. The Greenhouse–Geisser correction was used when the sphericity assumption did not hold. The normality of model residuals was additionally assessed using the Shapiro–Wilk test and graphical inspection of the residual distributions.

Since there were no missing data during the follow-up period, complete-case analysis was carried out. No prespecified subgroup or sensitivity analyses were conducted. To account for potential confounding inherent to the observational study design, an additional multivariable longitudinal analysis was performed using generalized estimating equations (GEE) with a Gaussian distribution and an exchangeable working correlation structure. Postoperative VAS scores were modeled as repeated measurements, with block group and postoperative time included as explanatory variables. Age, body mass index, operative duration, and ASA physical status were included a priori as clinically relevant potential confounders. Adjusted between-group differences are reported with corresponding 95% confidence intervals.

For all analyses, two-tailed *p* values less than 0.05 were regarded as statistically significant.

## 3. Results

### 3.1. Participant Flow

**Figure 1:**
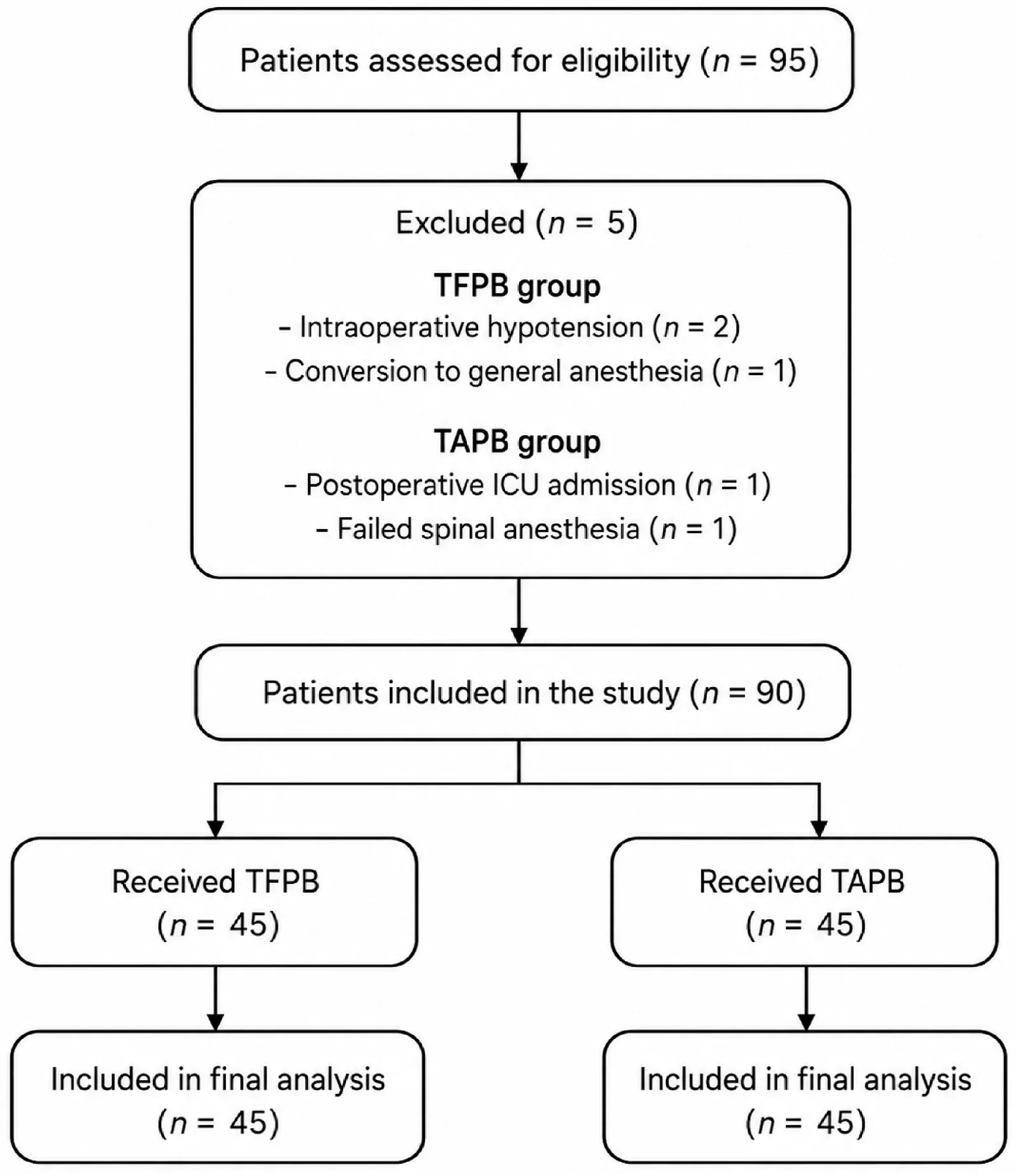
STROBE flowchart illustrating participant recruitment, allocation, follow-up, and inclusion in the final analysis.

The eligibility of 95 patients was evaluated. We excluded three patients from the TFPB group (two because of intraoperative hypotension and one because of conversion to general anesthesia) and two patients from the TAPB group (one because of postoperative intensive care unit admission and one because of unsuccessful spinal anesthesia), constituting the five patients who were excluded from the study. Finally, 90 patients undergoing elective repeat cesarean delivery were included in the final analysis. A bilateral ultrasound-guided transversalis fascia plane block (Group TFPB) was administered to 45 patients, while a bilateral ultrasound-guided transversus abdominis plane block (Group TAPB) was administered to a different set of 45 patients. All participants had access to complete postoperative follow-up data. A flowchart summarizing patient enrollment and the course of the study is given in Figure 1.

### 3.2. Baseline Characteristics

The two groups’ baseline demographic and perioperative characteristics were similar. Patient characteristics and perioperative variables are summarized in Table 1. Age, body mass index, ASA physical status, and length of operation did not differ statistically significantly between the groups. The mean cesarean delivery number was comparable between the TFPB and TAPB groups (2.41 ± 0.19 vs. 2.49 ± 0.23, *p* = 0.489).

### 3.3. Primary Outcomes

#### 3.3.1. Resting VAS Scores

**Figure 2:**
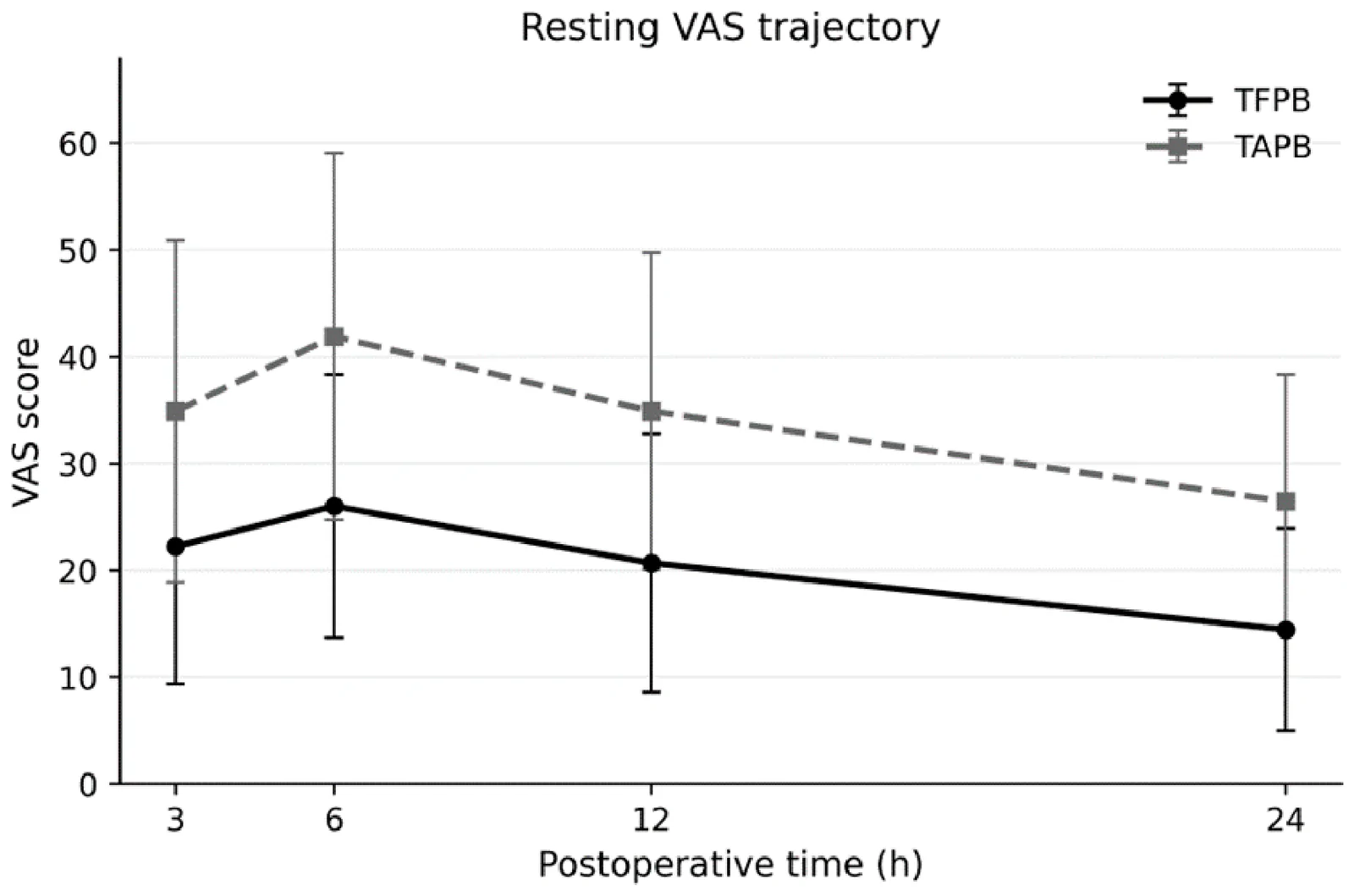
Changes in resting postoperative VAS scores over the first 24 h after surgery in the TFPB and TAPB groups. Error bars indicate the standard deviation. Overall between-group effect (mixed ANOVA): *p* < 0.001. VAS, Visual Analog Scale (0–100 mm).

For resting VAS ratings, a mixed-design repeated-measures ANOVA showed significant group, time, and group-by-time interaction effects (all *p* < 0.001). Throughout the first 24 h following surgery, the TFPB group’s resting VAS scores were consistently lower than those of the TAPB group. Serial resting VAS scores are illustrated in Figure 2.

The mean resting VAS score over the first 24 postoperative hours was lower in the TFPB group than in the TAPB group (20.83 ± 8.21 vs. 34.53 ± 10.14), with a mean between-group difference of 13.69 mm (95% CI, 9.83–17.56; *p* < 0.001).

#### 3.3.2. Movement VAS Scores

**Figure 3:**
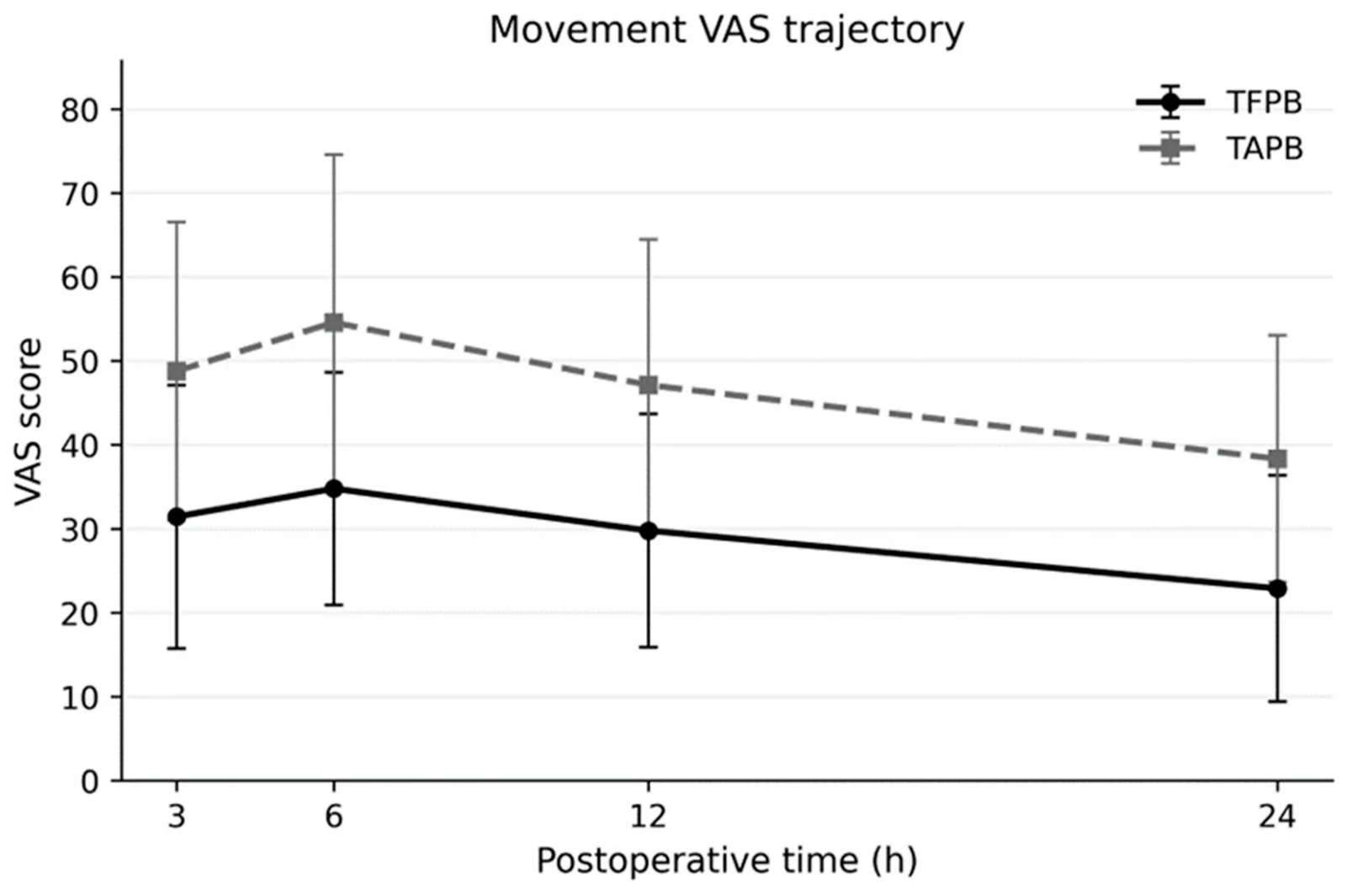
Changes in postoperative VAS scores during movement over the first 24 h after surgery in the TFPB and TAPB groups. Error bars indicate the standard deviation. Overall between-group effect (mixed ANOVA): *p* < 0.001.

Analysis of repeated movement-related VAS measurements using mixed-design ANOVA demonstrated significant effects of treatment group and postoperative time as well as significant interaction between these factors (all *p* < 0.001). Across all postoperative assessments, patients in the TFPB group reported lower movement VAS scores than those in the TAPB group. The temporal pattern of movement VAS scores is illustrated in Figure 3.

The mean movement-related VAS score over the first 24 postoperative hours was lower in the TFPB group than in the TAPB group (29.72 ± 10.59 vs. 47.19 ± 12.88), with a mean between-group difference of 17.47 mm (95% CI, 12.53–22.41; *p* < 0.001). In the adjusted longitudinal analysis, block type remained associated with postoperative pain scores after adjustment for age, BMI, operative duration, and ASA physical status. Compared with TFPB, TAPB was associated with an adjusted increase of 14.06 mm in resting VAS scores (95% CI, 10.05–18.07; *p* < 0.001) and 17.91 mm in movement VAS scores (95% CI, 12.64–23.18; *p* < 0.001). Residual diagnostics for the repeated-measures ANOVA indicated some departure from strict normality (Shapiro–Wilk *p* < 0.001 for resting VAS and *p* = 0.046 for movement VAS). Accordingly, strict residual normality could not be assumed. However, the additional longitudinal GEE analyses yielded consistent between-group associations after adjustment for clinically relevant potential confounders.

The observed Pearson correlations among repeated resting VAS measurements ranged from 0.089 to 0.640, with a mean pairwise correlation of 0.426. For movement VAS measurements, correlations ranged from 0.283 to 0.739, with a mean pairwise correlation of 0.533. Thus, the overall observed correlation structure was reasonably consistent with the correlation coefficient of 0.50 assumed in the a priori sample size calculation.

Figure 2 and Figure 3 show the VAS measurements regarding resting and moving repeatedly after surgery, which are summarized in Table 2.

### 3.4. Secondary Outcomes

#### 3.4.1. Mobilization Outcomes

**Figure 4:**
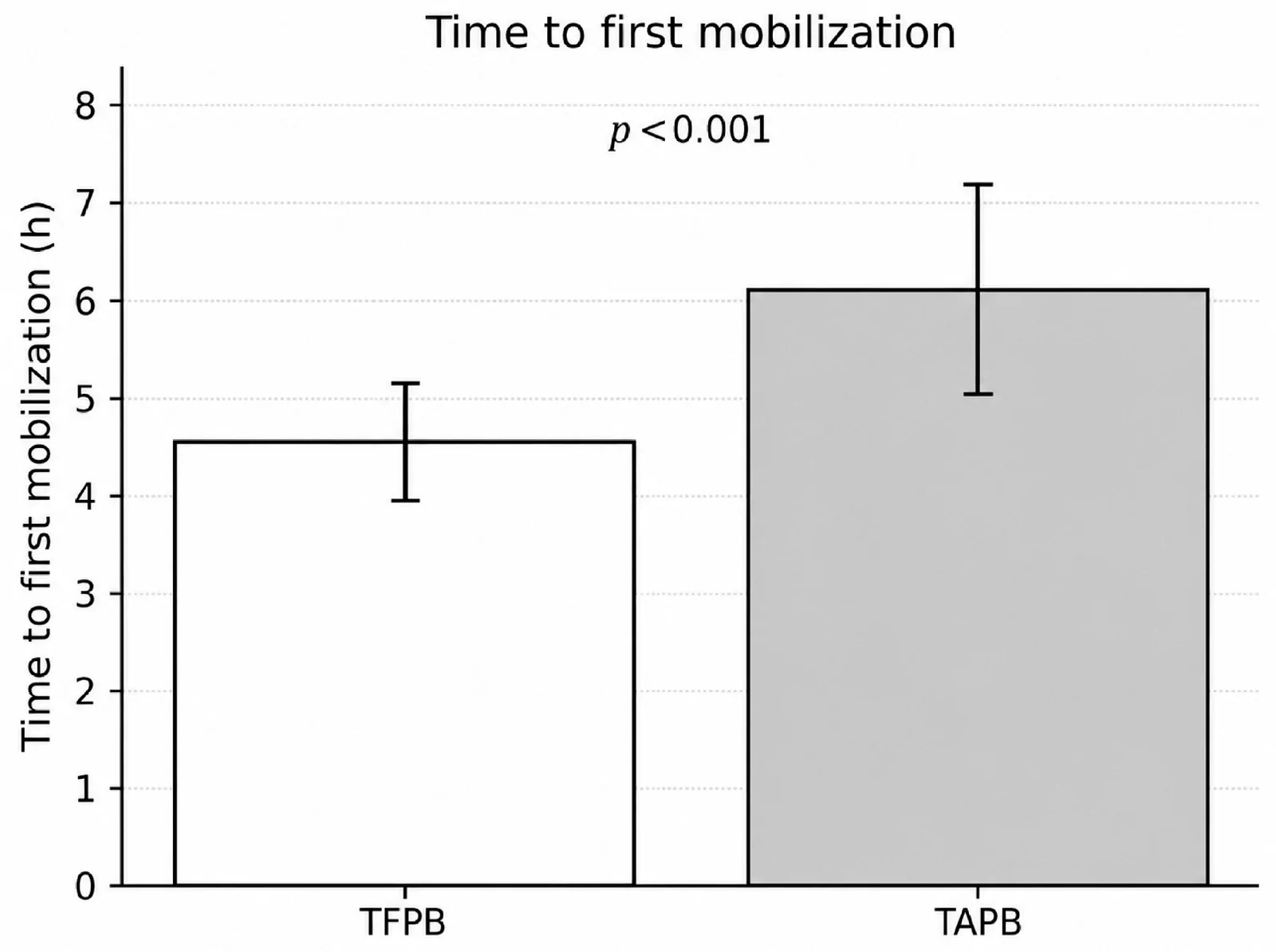
Comparison of time until first mobilization between groups. Error bars indicate the standard deviation.

Patients in the TFPB group achieved first mobilization earlier than those in the TAPB group (4.55 ± 0.62 h vs. 6.11 ± 1.07 h). The mean between-group difference in time to first mobilization was 1.56 h (95% CI, 1.19–1.93; *p* < 0.001). These findings are illustrated in Figure 4.

#### 3.4.2. Analgesic Consumption

Requirements for additional postoperative analgesics beyond the standardized baseline analgesic regimen were lower in the TFPB group. Additional paracetamol consumption during the first 24 postoperative hours was significantly lower in the TFPB group than in the TAPB group (866.7 ± 694.1 mg vs. 1711.1 ± 920.0 mg). The estimated mean difference between groups was 844.44 mg (95% CI, 502.65–1186.23; *p* < 0.001).

The mean between-group difference in additional diclofenac sodium consumption was 36.67 mg (95% CI, 20.33–53.00; *p* < 0.001). The mean between-group difference in additional tramadol consumption was 11.11 mg (95% CI, −2.00 to 24.23; *p* = 0.101).

Rescue tramadol was administered to 11 of 45 patients (24.4%) in the TFPB group and 19 of 45 patients (42.2%) in the TAPB group.

**Figure 5:**
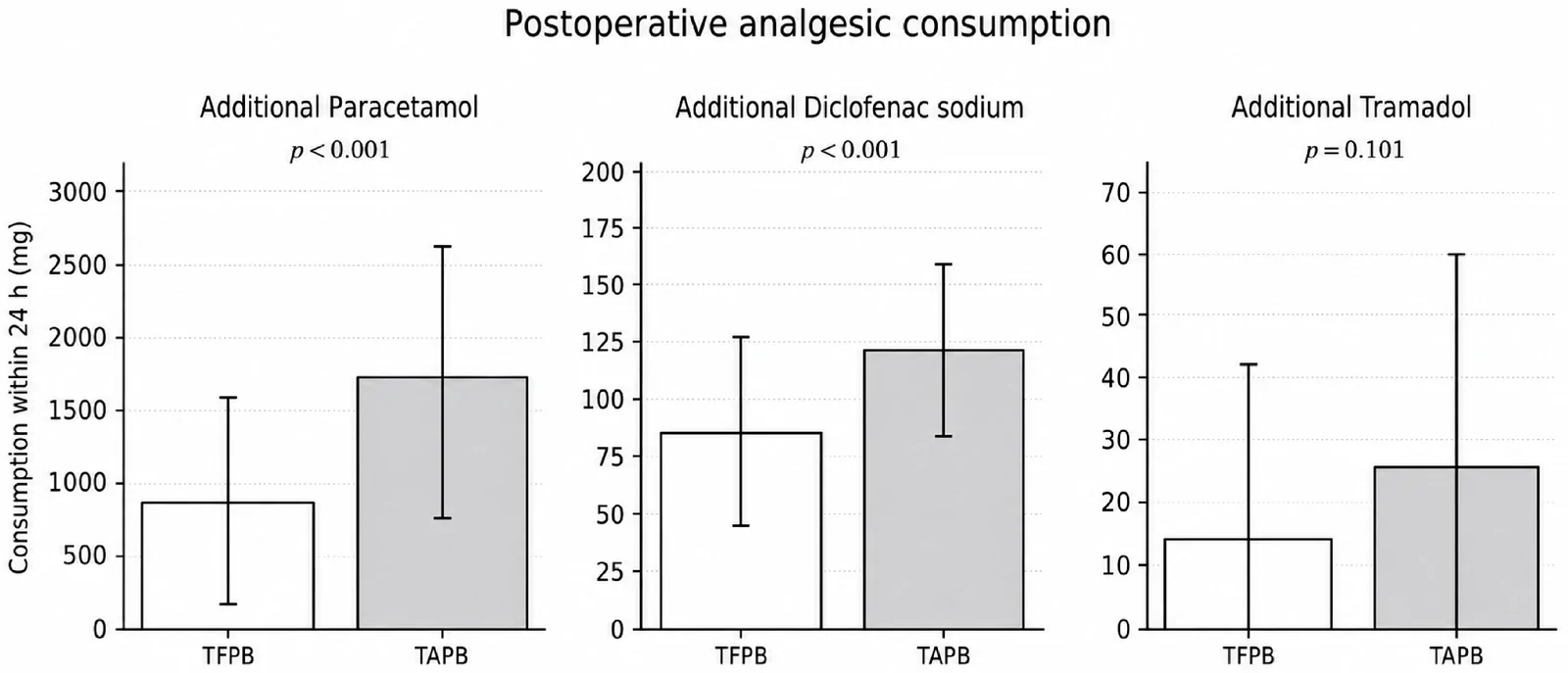
Comparison of additional postoperative analgesic consumption between groups. Error bars indicate the standard deviation.

Figure 5 summarizes the additional postoperative analgesic consumption. Additional tramadol requirements were also lower in the TFPB group, although the difference did not reach statistical significance. Mobilization outcomes and postoperative analgesic consumption are summarized in Table 3.

#### 3.4.3. Adverse Effects

The frequency of opioid-related adverse effects, including nausea, vomiting, pruritus, and dizziness, did not differ significantly between the TFPB and TAPB groups.

Detailed data regarding perioperative blood loss and perioperative hemoglobin measurements are presented in Supplementary Table S1. No intraoperative organ injuries occurred in either group.

## 4. Discussion

Among patients undergoing repeat cesarean delivery, TFP blocks were linked to lower postoperative pain levels, lower requirements for additional postoperative analgesics, and quicker mobilization relative to TAP blocks in this prospective observational analysis. Notably, the analgesic advantage of the TFPB persisted throughout the first postoperative day, with lower VAS scores observed during rest and mobilization at each evaluation time point.

Taken together, our results add to the accumulating evidence supporting the integration of ultrasound-guided fascial plane techniques into postoperative analgesic protocols for cesarean delivery [5,7,8]. Prior research assessing TAP blocks has shown decreases in postoperative pain scores and narcotic requirements following a cesarean section [9,10]. However, the analgesic efficacy of a TAP block may vary depending on the extent of local anesthetic spread and the dermatomal coverage achieved [11,12]. Our findings are also consistent with recent systematic reviews and meta-analyses demonstrating that TFPB reduces postoperative opioid consumption and rescue analgesic requirements after cesarean delivery compared with conventional analgesic strategies. Nevertheless, evidence directly comparing TFPB with TAPB remains limited, particularly in women undergoing repeat cesarean deliveries [16,17].

The TFPB group consistently had lower resting and movement VAS scores during all postoperative evaluation periods when postoperative pain outcomes were assessed longitudinally. Additionally, the TFPB group’s faster mobilization and decreased postoperative analgesic intake may have clinical significance in the context of Enhanced Recovery After Surgery (ERAS) pathways for cesarean delivery [6]. Among obstetric patients, early mobilization is especially crucial since delayed mobilization might worsen maternal functional recovery while increasing postoperative discomfort and thromboembolic risk [4,18,19].

The observed between-group difference in movement-related pain also appears to be clinically meaningful. The mean difference in movement VAS scores exceeded the commonly accepted minimum clinically important difference (MCID) of approximately 10 mm for acute postoperative pain, suggesting that the observed improvement is likely to be perceptible and relevant to patients [20,21]. Likewise, the approximately 1.5 h reduction in time to first mobilization may have practical importance within Enhanced Recovery After Surgery (ERAS) pathways by facilitating earlier functional recovery, maternal–newborn interaction, and postoperative ambulation. Furthermore, the observed correlations among repeated VAS measurements were broadly consistent with the correlation assumed in the a priori sample size calculation, and the 95% confidence intervals around the principal between-group estimates provided reasonably precise estimates of the magnitude and direction of the observed associations.

More proximal local anesthetic diffusion along the thoracolumbar fascial plane and improved blocking of T12–L1 nerve branches involved in Pfannenstiel incision pain transmission may be linked to the better analgesic profile seen with the TFPB. Compared with the TAP block, TFP blocks may provide superior coverage of the iliohypogastric and ilioinguinal nerves, which contribute substantially to lower abdominal incision pain after cesarean delivery [11,12].

Additionally, our results align with prior comparative studies comparing TFP and TAP blocks in lower-abdominal surgery and cesarean birth. Previous studies have suggested that TFPB provides effective postoperative analgesia after cesarean delivery, although direct comparisons with TAPB have yielded heterogeneous findings. More recent randomized trials and systematic reviews support the analgesic efficacy of TFPB, particularly regarding postoperative opioid consumption and rescue analgesic requirements, while emphasizing the need for further comparative studies [14,15,16,17,22,23].

The use of a homogeneous patient population entirely composed of repeat cesarean deliveries, standardized spinal anesthesia techniques, skilled block practitioners, thorough postoperative follow-ups, and repeated longitudinal pain assessments during the first 24 postoperative hours are some of this study’s strengths.

It should also be noted that intrathecal morphine was not used in our study, whereas it represents standard postoperative analgesic practice in many institutions. Therefore, the magnitude of the analgesic differences observed between TFPB and TAPB may differ in settings where intrathecal morphine or more intensive multimodal analgesic protocols are routinely employed. Consequently, the generalizability of our findings should be interpreted within the context of postoperative analgesic strategies similar to those used in the present study. This interpretation is consistent with the most recent PROSPECT recommendations for cesarean delivery, which recognize fascial plane blocks as appropriate adjuncts when long-acting neuraxial opioids are not administered [24].

The findings of this study may have important clinical implications for postoperative pain management following repeat cesarean delivery. In institutions where intrathecal opioids are not routinely administered, TFPB may represent an effective component of multimodal analgesia by reducing postoperative pain intensity and additional analgesic requirements and facilitating earlier mobilization. However, given the observational design of the present study, these findings should be interpreted cautiously and considered hypothesis-generating. Confirmation through adequately powered multicenter randomized controlled trials is warranted before these findings can be translated into routine clinical practice.

Several limitations should be acknowledged. First, because treatment allocation was based on anesthesiologist preference rather than randomization, selection bias and residual confounding cannot be excluded despite the comparable measured baseline characteristics between groups. Therefore, the observed differences should be interpreted as associations rather than evidence of a causal treatment effect. Second, as this study was conducted at a single institution, the generalizability of the findings to other clinical settings may be limited. Third, although postoperative outcome assessments were performed by independent anesthesiologists, the study was observational, and patients were not blinded to the intervention. Consequently, performance bias and detection bias, particularly for subjective outcomes such as VAS pain scores, cannot be completely excluded. Fourth, postoperative sensory mapping was not performed; therefore, the dermatomal distribution and successful sensory coverage of each fascial plane block could not be objectively confirmed. Consequently, individual variations in block spread or block success may have influenced the observed postoperative analgesic outcomes. Future studies incorporating standardized postoperative sensory assessments would help to better characterize block performance and its relationship with clinical outcomes. Finally, long-term postoperative pain outcomes and patient satisfaction measures were not evaluated.

However, these results may be applicable in comparable obstetric anesthetic settings owing to the uniform research population and consistent perioperative care.

## 5. Conclusions

In this single-center prospective observational study, TFP block was associated with lower postoperative pain scores, lower additional postoperative analgesic requirements, and earlier mobilization than TAP block in women undergoing repeat cesarean delivery. These findings should be interpreted as associations rather than evidence of a causal treatment effect. Confirmation of these observations will require well-designed multicenter randomized controlled trials.

## Figures and Tables

**Table 1:** Patient characteristics and perioperative variables.

Variable	TFPB (*n* = 45)	TAPB (*n* = 45)	*p* Value
Age, years	27.53 ± 3.95	28.58 ± 5.89	0.327
Height, cm	162.56 ± 5.07	162.93 ± 5.05	0.724
Weight, kg	75.78 ± 13.52	74.89 ± 10.61	0.730
BMI, kg/m^2^	28.72 ± 5.08	28.17 ± 3.48	0.552
ASA physical status (I/II), *n*	36/9	34/11	0.617
Operative duration, min	50.11 ± 11.89	47.44 ± 11.21	0.250
Cesarean delivery number	2.41 ± 0.19	2.49 ± 0.23	0.489

Data are presented as means ± standard deviations unless otherwise specified.

**Table 2:** Serial postoperative resting and movement VAS scores.

Time Point	TFPB (*n* = 45)	TAPB (*n* = 45)
Resting VAS—3 h	22.22 ± 12.86	34.89 ± 16.00
Movement VAS—3 h	31.44 ± 15.69	48.78 ± 17.74
Resting VAS—6 h	26.00 ± 12.32	41.89 ± 17.16
Movement VAS—6 h	34.78 ± 13.85	54.56 ± 19.99
Resting VAS—12 h	20.67 ± 12.08	34.89 ± 14.82
Movement VAS—12 h	29.78 ± 13.89	47.11 ± 17.34
Resting VAS—24 h	14.44 ± 9.48	26.44 ± 11.85
Movement VAS—24 h	22.89 ± 13.46	38.33 ± 14.69

VAS, Visual Analog Scale (0–100 mm), with higher scores indicating greater pain intensity. Values are expressed as means ± standard deviations. Inferential analysis of the longitudinal VAS trajectories was performed using mixed-design repeated-measures ANOVA; individual time-point *p* values are therefore not reported.

**Table 3:** Mobilization and postoperative analgesic consumption.

Variable	TFPB (*n* = 45)	TAPB (*n* = 45)	*p* Value
Time to first mobilization, h	4.55 ± 0.62	6.11 ± 1.07	*p* < 0.001
Additional Paracetamol consumption, mg	866.67 ± 694.13	1711.11 ± 920.03	*p* < 0.001
Additional Diclofenac sodium consumption, mg	85.00 ± 41.07	121.67 ± 36.77	*p* < 0.001
Additional Tramadol consumption, mg	14.44 ± 27.43	25.56 ± 34.74	*p* = 0.101

Values are expressed as means ± standard deviations (SDs). Analgesic consumption represents additional analgesics administered beyond the standardized baseline postoperative analgesic regimen. Abbreviations: TFPB, transversalis fascia plane block; TAPB, transversus abdominis plane block.

## Data Availability

The datasets generated and analyzed during the current study are available from the corresponding author upon reasonable request.

## Supplementary Materials

The following supporting information can be downloaded at: https://www.mdpi.com/article/10.3390/healthcare14172682/s1, Table S1: Comparison of perioperative blood loss and perioperative hemoglobin measurements between the TFPB and TAPB groups.

## Abbreviations

The following abbreviations are used in this manuscript:

ASA	American Society of Anesthesiologists
BMI	Body Mass Index
ERAS	Enhanced Recovery After Surgery
SPSS	Statistical Package for the Social Sciences
STROBE	Strengthening the Reporting of Observational Studies in Epidemiology
TAP	Transversus Abdominis Plane
TAPB	Transversus Abdominis Plane Block
TFP	Transversalis Fascia Plane
TFPB	Transversalis Fascia Plane Block
VAS	Visual Analog Scale
CI	Confidence Interval
