# Supplementary material for: Ultrasound-Guided Transversalis Fascia Plane Block Versus Transversus Abdominis Plane Block for Postoperative Analgesia After Repeat Cesarean Delivery: A Prospective Observational Study
Source: Healthcare (Basel). 2026 Aug 24;14(17):2682. doi: 10.3390/healthcare14172682 (PMC13564664; doi:10.3390/healthcare14172682)
Supplement: Supplementary file 1 [file healthcare-14-02682-s001.zip › Supplementary Table S1.pdf]

Supplementary Table S1. Comparison of perioperative blood loss and perioperative hemoglobin measurements between the TFPB and TAPB groups.

**Variable**	**TFPB (n=45)**	**TAPB (n=45)**	**p value**
**Preoperative hemoglobin (g/dL)**	11.83 ± 1.49	11.98 ± 1.50	0.644
**Postoperative hemoglobin (g/dL)**	10.36 ± 1.35	10.42 ± 1.32	0.838
**Hemoglobin decrease (%)**	12.29 ± 5.36	12.85 ± 5.22	0.617
**Estimated intraoperative blood loss (mL)**	290 ± 94.5	271.11 ± 63.5	0.445

Data are presented as mean ± standard deviation (SD). Median (minimum–maximum) values are available upon request. Group comparisons were performed using the Mann–Whitney U test where appropriate.
